# Root-derived cytokinin regulates Arabidopsis flowering time through components of the age pathway

**DOI:** 10.1093/plphys/kiaf204

**Published:** 2025-06-25

**Authors:** Isabel Bartrina, Sören Werner, Andreas Schenke, Debora Gasperini, Tomáš Werner, Thomas Schmülling

**Affiliations:** Institute of Biology/Applied Genetics, Dahlem Centre of Plant Sciences (DCPS), Freie Universität Berlin, Albrecht-Thaer-Weg 6, Berlin D-14195, Germany; Department of Biology, University of Graz, Schubertstraße 51, Graz A-8010, Austria; Institute of Biology/Applied Genetics, Dahlem Centre of Plant Sciences (DCPS), Freie Universität Berlin, Albrecht-Thaer-Weg 6, Berlin D-14195, Germany; Department of Molecular Signal Processing, Leibniz Institute of Plant Biochemistry, Weinberg 3, Halle D-06120, Germany; Department of Molecular Signal Processing, Leibniz Institute of Plant Biochemistry, Weinberg 3, Halle D-06120, Germany; Department of Biology, University of Graz, Schubertstraße 51, Graz A-8010, Austria; Institute of Biology/Applied Genetics, Dahlem Centre of Plant Sciences (DCPS), Freie Universität Berlin, Albrecht-Thaer-Weg 6, Berlin D-14195, Germany

## Abstract

The transition to flowering is governed by different pathways integrating endogenous and exogenous signals. Here, we evaluated the role of the phytohormone cytokinin (CK) in regulating *Arabidopsis thaliana* flowering time. By analyzing key mutants in CK metabolism, transport, and signaling, we found that the hormone promotes flowering under both long-day (LD) and short-day (SD) conditions, with a stronger impact on flowering under SDs. Genetic analyses indicated that both trans- and cis-zeatin regulate the floral transition, while isopentenyladenine plays a minor role. Blocking CK export from roots and reciprocal grafting experiments revealed that root-derived CK is an important flowering signal. Perception and transmission of the CK flowering signal depended on distinct CK receptors, phosphotransmitter proteins and several B-type response regulators. Further, CK functioned through floral integrators such as SUPPRESSOR OF OVEREXPRESSION OF CO1 (SOC1) and components of the age pathway. The CK status of plants affected the levels of the age pathway microRNAs miR156 and miR172. Cytokinin-promoted flowering required the miR156-target *SQUAMOSA PROMOTER BINDING PROTEIN-LIKE15* (*SPL15*) and miR172, and the late-flowering phenotype of LD-grown CK-deficient plants depended on miR172-targeted *APETALA2* (*AP2*)*-like* genes encoding floral repressors. Collectively, this study shows that CK regulates flowering time through the two-component signaling system and components of the age pathway, providing a genetic framework for future investigations.

## Teaser

Root-borne phytohormone cytokinin regulates flowering time through distinct elements of the two-component signaling pathway, components of the age pathway, and key regulators of the floral transition.

## Introduction

When to flower is one of the most important decisions during a plant’s life, having a decisive bearing on its reproductive success and the survival of its offsprings. Studies in the model plant *Arabidopsis thaliana* have identified six different pathways regulating the transition to flowering, and integrating environmental conditions (light, temperature) with endogenous factors such as hormones or plant age (Srikanth and Schmid 2011; Turnbull 2011; Andrés and Coupland 2012). The photoperiod pathway responds to day length and is the dominant pathway under long-day (LD) conditions. The vernalization pathway responds to low temperature over long periods and the ambient temperature pathway mediates changes in daily growth temperatures. The autonomous pathway accelerates flowering independently of day length and the gibberellin pathway defines a requirement for gibberellin for flowering. Finally, the age pathway depends on the temporally antagonistic expression patterns of two microRNAs, miR156 and miR172. These act sequentially to regulate the acquisition of flowering competence and the transition to flowering (Wang et al. 2009; Wu et al. 2009; Wang 2014).

The input from the different flowering pathways is integrated by a core set of floral pathway integrator genes including *FD*, *FLOWERING LOCUS T* (*FT*) and *SOC1*, which all promote floral development (Srikanth and Schmid 2011). FT and its paralogue TWIN SISTER OF FT (TSF) are mobile signals originating from the leaves (Yamaguchi et al. 2005; Corbesier et al. 2007). They act together with FD in the shoot apical meristem (SAM) to activate transcription of *SOC1* (Abe et al. 2005).

In addition to these well-established pathways, the hormone cytokinin (CK) has been long considered as one of the prime candidates for being (part of) the leaf-derived floral inducer florigen and/or to be a root-borne signal regulating flowering time (Bernier and Perilleux 2005). The first report on a possible role of CK in regulating flowering time has been published by Michniewicz and Kamieńska (1965) who described that treatment of *Arabidopsis* plants grown under SD conditions with the artificial CK kinetin induced flowering. In *Sinapis alba* CK induces expression of the *SOC1* homolog *SaMADS A* (Bonhomme et al. 2000). In both *Arabidopsis* and lychee, endogenous CK levels increase in leaves and phloem sap concomitantly with floral initiation (Corbesier et al. 2003; Stern et al. 2003). In addition, exogenous CK applied to the roots induces flowering in *Arabidopsis* under SD conditions (D'Aloia et al. 2011). However, a systematic genetic analysis, the identification of the CK genes involved in this process and the link(s) to the above-described flowering pathways is missing.

The plant hormone CK orchestrates numerous developmental and physiological processes (Werner and Schmülling 2009; Kieber and Schaller 2018; Argueso and Kieber 2024). The rate-limiting step in CK biosynthesis is catalyzed by isopentenyltransferases (IPTs) resulting in the formation of isopentenyladenine (iP) ribotide (Kakimoto 2001; Takei et al. 2001). This can be subsequently converted by hydroxylation of the isoprenoid side chain catalyzed by a pair of cytochrome P450 enzymes, CYP735A1 and CYP735A2, which are active mainly in the root, to trans-zeatin (*t*Z) ribotide (Takei et al. 2004). CK ribotides are activated by conversion into the corresponding free bases iP and *t*Z, by the LONELY GUY (LOG) family of enzymes (Kuroha et al. 2009; Tokunaga et al. 2012). The levels of active CK in a cell can be decreased either through conjugation to glucose or through irreversible cleavage by CK oxidase/dehydrogenases (CKXs) (Mok and Mok 2001; Werner et al. 2006).

CK can be synthesized and catabolized in a variety of tissues, including roots, leaves, vasculature, and the SAM. The hormone may fulfill local functions, but it may also be transported elsewhere in the plant, with both leaves and roots acting as potential CK sources. The iP-type CKs move rootward in the phloem (Matsumoto-Kitano et al. 2008; Bishopp et al. 2011), while the *t*Z-type CKs move shootward in the xylem mainly as ribosides but also as free bases (Kiba et al. 2013; Osugi et al. 2017). Crucial for the root export of *t*Z-type CKs is a member of the subfamily G of ATP-BINDING CASSETTE proteins, ABCG14 (Ko et al. 2014; Zhang et al. 2014).

The CK signal is perceived and transduced by a histidine kinase phosphorelay system (Müller and Sheen 2007). In *Arabidopsis*, there are three membrane-spanning histidine protein kinases (AHKs) that serve as CK receptors, AHK2, AHK3, and AHK4/CYTOKININ RESPONSE1 (CRE1) (Inoue et al. 2001; Ueguchi et al. 2001; Yamada et al. 2001) and signal via histidine phosphotransfer proteins (AHPs) to type-B response regulators (RRBs), which are transcription factors (Müller and Sheen 2007; Leuendorf and Schmülling 2021). Among others they activate expression of genes encoding type-A response regulators (RRAs), which act as negative regulators of CK signaling.

CK-deficient *Arabidopsis* plants (overexpressing the CK-degrading enzyme CKX1) flower later under LD conditions and do not flower at all under SD conditions (Werner et al. 2003; Bartrina et al. 2017). Furthermore, *repressor of cytokinin deficiency2* (*rock2*) mutants harboring a constitutively active gain-of-function variant of the CK receptor AHK2 flower earlier under LDs and SDs (Bartrina et al. 2017). Other previous work has occasionally reported altered flowering time of CK mutants or transgenic lines with an altered CK content (Nishimura et al. 2004; Ishida et al. 2008; Werner et al. 2010, 2021a; Holst et al. 2011; Tokunaga et al. 2012; Kiba et al. 2013). The available genetic data support a promotive activity of CK in regulating flowering time.

In the age pathway, primary miR156 transcripts are produced from ten different *MIR156* loci, *MIR156A* to *MIR156J* (Breakfield et al. 2012). Mature miR156 suppresses several *SPL* genes by transcript cleavage and translational repression. miR172 acts downstream of miR156 and is also produced from several genetic loci, *MIR172A* to *MIR172E*, which are direct targets of SPL transcription factors (Wang et al. 2009; Xu et al. 2016). miR172 acts primarily through translational inhibition of *AP2* and *AP2*-like transcription factor genes *SCHLAFMUETZE* (*SMZ*), *SCHNARCHZAPFEN* (*SNZ*), and *TARGET OF EAT1* (*TOE1*), *TOE2*, and *TOE3*, which all encode redundantly acting negative regulators of flowering (Aukerman and Sakai 2003; Schmid et al. 2003).

miR156 levels are high in seedlings and decrease as plants age, while the target *SPL* transcript abundances exhibit an opposite behavior (Wang et al. 2009; Xu et al. 2016). miR172 levels are low during early plant development and increase with age, with the consequential decrease of its targets (Mathieu et al. 2009). Mutation or overexpression of miR156, miR172 and their target genes alter flowering time. Low miR156 expression levels are required for transition to flowering and lowering the miR156 activity by expression of a target mimicry gene (*MIM156*) causes early flowering under both LD and SD (Wang et al. 2009). High expression of miR156 prevents precocious flowering in young plants and causes late flowering irrespective of day length (Schwab et al. 2005; Wang et al. 2009; Wu et al. 2009). Conversely, high miR172 expression accelerates the transition to flowering under both SD and LD (Aukerman and Sakai 2003) and mutation of the *MIR172A*, *MIR172B* and *MIR172D* genes delays flowering under SD conditions (Lian et al. 2021; O'Maoileidigh et al. 2021). Mutation of miR172 target genes causes early flowering and *SMZ* or *SNZ* overexpression causes late flowering, documenting their role as repressors of the floral transition (Aukerman and Sakai 2003; Mathieu et al. 2009). Taken together, the miR156–SPL and miR172–AP2 modules are central to regulate acquisition of competence to flower and to coordinate the transition to flowering (Huijser and Schmid 2011; Hyun et al. 2017).

Here we describe the systematic analysis of flowering behavior of numerous CK metabolism, transport and signaling mutants and identify core genes of the CK flowering pathway. Excitingly, root-derived CK has an important role in regulating floral transition in particular under SD conditions. We show that CK-induced flowering requires the floral integrators SOC1 and FD-FT/TSF and it is mediated, at least in part, through components of the age pathway.

## Results

### CK promotes flowering under LD and SD conditions

**Figure 1.**
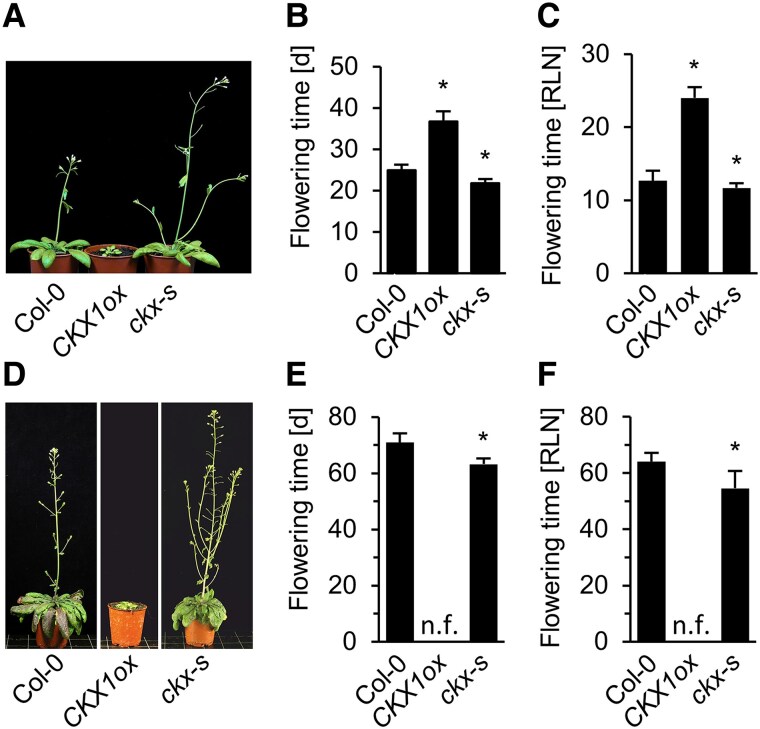
CK promotes flowering under SD and LD conditions. Flowering phenotypes of *CKX1ox* and *ckx-s* under LD **A** to **C)** and SD **D** to **F)** conditions as compared to WT (Col-0). The diameter of the pots in (A) and (D) is 6 cm. d, days after sowing; RLN, rosette leaf number; n.f., not flowering. Data presented in B, C, E and F are means ± SD (*n* = 10). The statistical significance of differences compared with WT grown under the same conditions was calculated by Kruskal–Wallis **B, C)** or Student’s *t*-test **E, F)**. * *P* < 0.01.

To evaluate the influence of CK on flowering time under different day lengths, we compared the flowering behavior of plants with a lower (*CKX1ox*) or higher (*CKX* sextuple mutant *ckx2,3,4,5,6,7*; named *ckx-s*) CK content under both LD and SD conditions. Flowering time was recorded as days to bolting and the number of rosette leaves at bolting. Under LD conditions, *CKX1ox* flowered about 12 d later than the wild type (WT), which started flowering after 25 ± 1.3 d (Fig. 1, A and B). *CKX1ox* plants developed 24 ± 1.5 leaves until bolting while WT developed 13 ± 1.4 leaves (Fig. 1C). In contrast, the *ckx-s* mutants flowered about 3 d earlier than WT in LDs and formed also less leaves until bolting (Fig. 1A to C). A stronger effect was observed under SD conditions. The CK-deficient *CKX1ox* plants remained in the vegetative stage even after 120 d consistent with earlier reports (Bartrina et al. 2017), wild-type plants flowered after 71 ± 3.3 d with 64 ± 3.1 leaves and *ckx-s* mutants flowered after 63 ± 2.0 d with 55 ± 6.2 leaves (Fig. 1D to F). The opposite behavior of *CKX1ox*, flowering later after having formed more leaves, and *ckx-s*, flowering earlier with less leaves, indicates that CK acts as a positive regulator of flowering time under both photoperiods.

**Figure 2.**
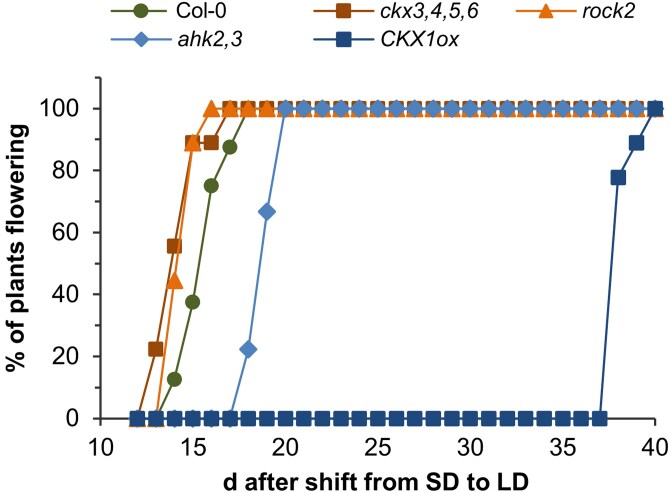
Induction of flowering after a shift from SD to LD conditions. All plants were grown under SD for 4 wk and then shifted to LD conditions. Results are shown as percentage of plants having started to flower after the shift to LD conditions (*n* > 8).

Next, we tested the response to a change in photoperiod of two prototypic examples of metabolism (*ckx3,4,5,6*; *CKX1ox*) and signaling (*rock2*; *ahk2 ahk3*) mutants with respectively higher and lower CK content or signaling by shifting 4-wk-old SD-grown plants to permissive LD conditions. The mutants showed similar differences in flowering time as under constant conditions. The two genotypes with a higher CK content (*ckx3,4,5,6*) or signaling (*rock2*) showed an earlier transition to flowering while the genotypes with a lower CK content (*CKX1ox*) or signaling (*ahk2,3*) started to flower later than WT (Fig. 2). This result confirmed the promotive effect of CK on the transition to flowering.

To identify CK metabolism and signaling genes that are relevant to regulate flowering time, a series of CK metabolism, transport and signaling mutants was tested under SD and LD conditions. We first analyzed the role of CK in more detail under SD conditions, where we observed a stronger effect and in which the promoting effect of the photoperiod pathway is absent. All mutants with a higher CK status (different *ckx* mutants*, rock2, rra* mutants; printed in red in Table 1) flowered up to about 13 d earlier or at a similar time as WT, and *ckx-s* as well as *rock2* formed less leaves until bolting (Table 1; Supplementary Fig. S1). In contrast, mutants with a lower CK status (*ipt, cyp735, log, ahk, ahp, rrb* mutants and *CKXox* plants; printed in blue in Table 1) flowered most clearly later than the WT, however with a varying number of leaves (Table 1; Supplementary Fig. S1).

**Table 1.** Flowering time of Arabidopsis CK metabolism and signaling mutants grown in SD.

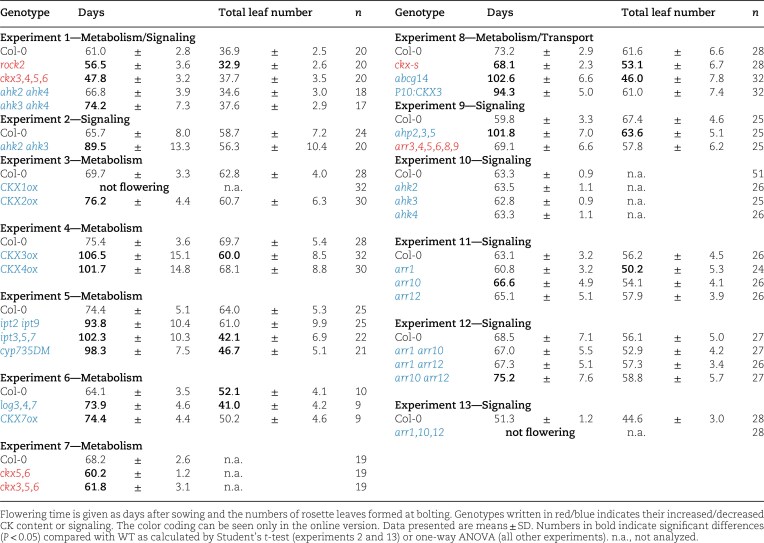

CK biosynthesis mutants having either a reduced concentration of iP- and/or *t*Z-type CK (*ipt3,5,7*; *log3,4,7*; *cypDM*) or lacking *c*Z-type CK (*ipt2,9*) flowered 20 to 30 d later than WT. This indicated that different types of CKs (iP-, *t*Z-, and *c*Z-type CKs) contribute to regulating flowering time (Table 1).

Mutation of cytokinin-degrading *CKX* genes caused earlier flowering compared with WT, while overexpression of different *CKX* genes with different substrate specificities and different subcellular locations (Werner et al. 2003; Galuszka et al. 2007; Köllmer et al. 2014; Niemann et al. 2015) retarded the transition to flowering, although to a different extent (Table 1). *CKX1ox* plants did not flower under SD conditions. Enhanced expression of either *CKX3* or *CKX4* caused about 30 d later flowering although their impact on shoot morphology is different. Overexpression of *CKX3* induced a strong reduction of rosette growth, while rosette growth of *CKX4* overexpressing plants was less affected (Werner et al. 2003) (Supplementary Fig. S1). Overexpression of *CKX2* and *CKX7*, the latter encoding the only CKX protein localized to the cytosol, had only a relatively mild effect on flowering time (Table 1). Together, the data showed a differential effect of *CKX* gene overexpression on flowering time and illustrated that the impact of CK on flowering time is not coupled to its effect on overall shoot growth.

Functional redundancy in regulating flowering time was also found among the CK signaling genes but there was also some specificity. Flowering time was retarded in the receptor double mutants *ahk2 ahk3* and *ahk3 ahk4*, but was similar to WT in the *ahk2 ahk4* mutant (Table 1; Supplemental Figure S1). As the flowering phenotype of the single *ahk2, ahk3* and *ahk4* mutants resembled the WT (Table 1; see also Nishimura et al., 2004 and Riefler et al., 2006), this indicates that the AHK3 receptor acts redundantly with each of the two other receptors in the CK flowering pathway, but is also alone fully sufficient to transmit a flowering signal. Notably, retardation of flowering in *ahk2 ahk3* and *ahk3 ahk4* was less strong than in several CK synthesis or transport mutants, indicating that the remaining AHK4/CRE1 or AHK2 receptors alone are able to transmit at least a weak flowering signal in these double mutants. Furthermore, flowering is strongly impaired in *ahk2,3,4* triple mutants (Higuchi et al. 2004; Nishimura et al. 2004; Riefler et al. 2006).

Mutations in components of the next signaling step (*AHP2*, *AHP3*, and *AHP5*) caused very late flowering, in line with the CK flowering signal being transmitted through the two-component signaling system (Table 1).

Comparison of several single and double *rrb* mutants showed slightly later flowering with a mutated *ARR10* allele (Table 1). The triple *arr1,10,12* mutant failed to flower until the end of the experiment after 146 d similar to *CKX1ox* (Table 1). Together, this analysis indicated functional redundancy among the transcription factor genes *ARR1*, *ARR10*, and *ARR12*, which is a common observation for their different activities (Argyros et al. 2008; Ishida et al. 2008).

The flowering behavior of the multiple type-A response regulator mutant *arr3,4,5,6,8,9* was similar to the WT indicating that these type-A ARRs have no apparent function in regulating flowering time under SD conditions (Table 1; Supplementary Fig. S1).

Analysis of a subset of these CK mutants was tested under under LD conditions. Generally, the results confirmed that CK promotes flowering also under LD condition; however, its impact is clearly lower than under SD conditions (Supplementary Table S1). Genotypes with an increased CK status flowered earlier than WT and *ckx-s* had formed less rosette leaves at the time of bolting (Fig. 1). In contrast, genotypes with a lowered CK status flowered significantly later than WT under LD conditions (Supplementary Table S1). For example, a strong retardation of flowering time was noted for *CKX1ox*, and the *ahp2,3,5* and *arr1,10,12* signaling mutants. *CKX1ox* formed also more rosette leaves than WT before starting to flower (Fig. 1). Thus the same combination of receptors, phosphotransmitter proteins and transcription factors that are functionally most relevant under SD also have central roles under LD.

### Leaf number and flowering time are not coupled in all CK mutants

One intriguing observation from the analysis of flowering time of different CK mutants (Table 1) was that despite the strong positive correlation of the plant CK status and plant age at the transition to flowering, the number of leaves at bolting did not correlate in all genotypes with flowering time. For example, the metabolism mutants *ipt3,5,7* and *cypDM* flowered considerably later than WT (102.3 ± 10.3 and 98.3 ± 7.5 d as compared with 74.4 ± 5.1 d) while they had formed many fewer leaves than WT at bolting time. The same observation was made for the CK transport mutant *abcg14* (Table 1). This disparity suggests that certain CK genes are involved in regulating the leaf plastochrone interfering with the measurement of flowering time in terms of leaf number. Indeed, these mutants would be rated as early flowering based on leaf number while actually onset of flowering was retarded. Therefore, to avoid interference from an independent trait regulated by CK, the plastochrone, and because of the very strong correlation between CK status and bolting time, we have recorded the time to bolting in days to determine flowering time instead of counting the number of leaves that had formed until then. Indeed, already Bernier and Perilleux (2005) had noted that CKs affect the rate of leaf initiation more than the flowering time which hampered the discovery of CK activity in regulating flowering. Genetic studies indicated that plastochrone and flowering time are regulated by at least partially different pathways (Stratton 1998; Pouteau et al. 2004; Mendéz-Vigo et al. 2010; Salomé et al. 2011), which might be affected differently by CK.

### CK is a root-derived regulator of flowering time

Several earlier studies suggested that CK may be stimulating flowering time by transclocating from root tissues (Bernier and Perilleux 2005; D'Aloia et al. 2011). We took advantage of several mutants and transgenic lines to address the question whether a reduced export of CK from roots would affect flowering.

The *cypDM* mutant lacking formation of *t*Z-type CK, which are mainly synthesized in roots (Kiba et al. 2013), and *abcg14* lacking export of *t*Z-type CK from roots (Ko et al. 2014; Zhang et al. 2014) showed both a strong delay in flowering in plants grown under SD and LD conditions (Table 1, Supplementary Table S1), supporting that root-derived CK promotes flowering. In addition, we analyzed *P10:CKX3* plants, which express *CKX3* under the transcriptional control of the predominantly root-expressed *PYK10* promoter of *Arabidopsis* causing an enhanced CK breakdown in roots (Werner et al. 2010). Despite the absence of obvious morphological changes in the shoot (Supplementary Fig. S1), *P10:CKX3* also showed delayed flowering under SD and LD conditions (Table 1, Supplementary Table S1).

**Figure 3.**
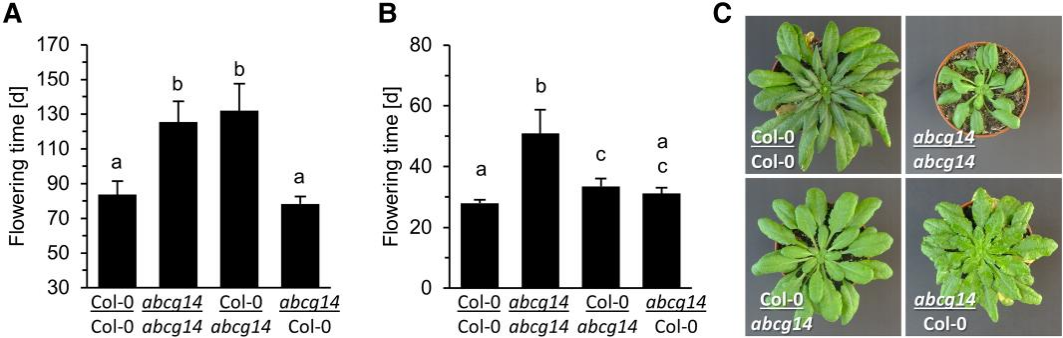
Root-derived CK regulates flowering time. **A, B)**, Flowering time of grafted plants of the indicated scion/rootstock genotype grown under SD (A) and LD (B) conditions. Data presented are means ± SD (*n* = 8–13). Letters shared between the genotypes indicate no significant difference (one-way ANOVA, *P* < 0.05). **C)**, Photograph of grafted plants grown under SDs at 80 DAG. The diameter of the pots is 6 cm.

The functional relevance of root-synthesized CK in regulating flowering time was further analyzed in reciprocal graft combinations between WT and the *abcg14* mutant. Figure 3 shows that self-grafted WT/WT and *abcg14*/*abcg14* plants reproduced the differences in flowering time found between ungrafted plants of the two genotypes under both SD and LD conditions. Under SD conditions, grafting WT scions on *abcg14* rootstocks (WT/*abcg14*) delayed the transition to flowering compared with WT/WT controls and started flowering as late as the *abcg14*/*abcg14* self-grafts (Fig. 3A). This delay was much less pronounced in WT/*abcg14* grafts grown under LD conditions (Fig. 3B). Conversely, WT rootstocks grafted to *abcg14* scions completely restored the late flowering of the mutant, with *abcg14*/WT plants flowering as early as WT/WT controls under both SD and LD conditions (Fig. 3). Collectively, these experiments unequivocally show that root-derived CK is a regulator of flowering time in *Arabidopsis* particularly under SD conditions.

### SOC1 is required for the flowering response to CK

**Figure 4.**
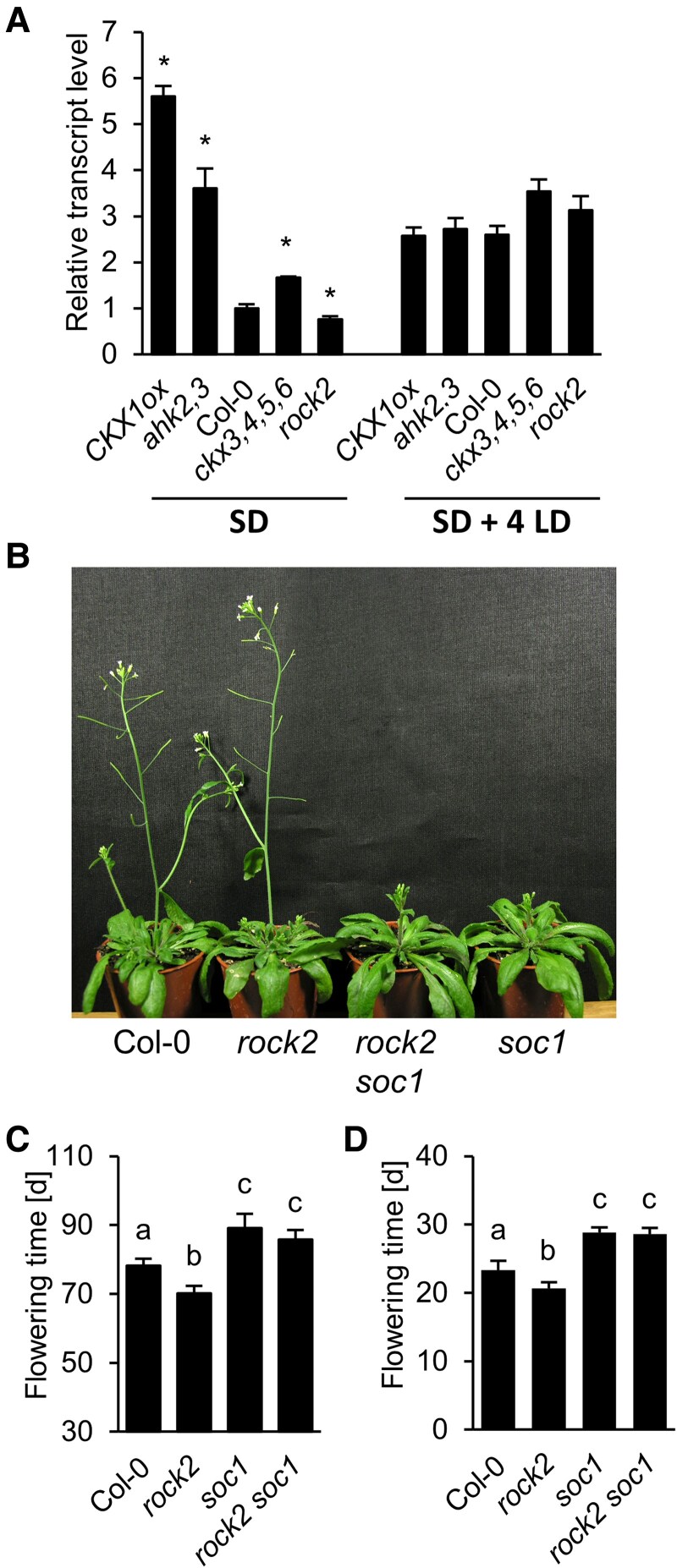
Analysis of *SOC1* expression and function in CK mutants. **A)** Relative abundance of *SOC1* transcripts in plants grown for 4 wk under SD conditions and after floral induction by four additional LD (SD + 4 LDs). Total RNA was extracted from hand-dissected shoot apices. Expression levels were normalized to *PP2AA2*. The transcript levels in Col-0 were set to 1. Data presented are means ± SD (*n* = 3). Stars indicate statistically significant differences to Col-0 (one-way ANOVA, *P* < 0.05). **B** to **D)**, Flowering time of WT, *rock2*, *soc1*, and *rock2 soc1* under SD (B, C) and LD (D) conditions. Data presented in C and D are means ± SD (*n* = 12). The diameter of the pots in (B) is 6 cm. Letters shared between the genotypes **C, D)** indicate no significant difference (one-way ANOVA, *P* < 0.05).

We then investigated which of the known floral integrator genes were required to mediate a response to CK. A first candidate gene was *SOC1*, a MADS box gene known to be upregulated by exogenous CK and which is required to promote flowering after CK treatment in SD (D'Aloia et al. 2011). Under SD, mutants with a low CK status unexpectedly showed a strongly increased level of *SOC1* transcript at different developmental stages (Fig. 4A, Supplementary Fig. S2A), although they flower late or not at all under these conditions (Table 1). The differences in *SOC1* transcript levels between WT and CK mutants disappeared after exposure of plants to flowering-inducing LD conditions (Fig. 4A). To assess the requirement of SOC1 for early flowering of *rock2* mutants, the flowering time of *soc1 rock2* double mutant was compared with the WT and single mutants. Absence of SOC1 abolished the early flowering phenotype of *rock2* under both SD and LD conditions (Fig. 4, B to D), demonstrating the requirement of SOC1 to mediate CK action on the transition to flowering under both photoperiods.

### CK regulates flowering through the FD-FT/TSF module

**Figure 5.**
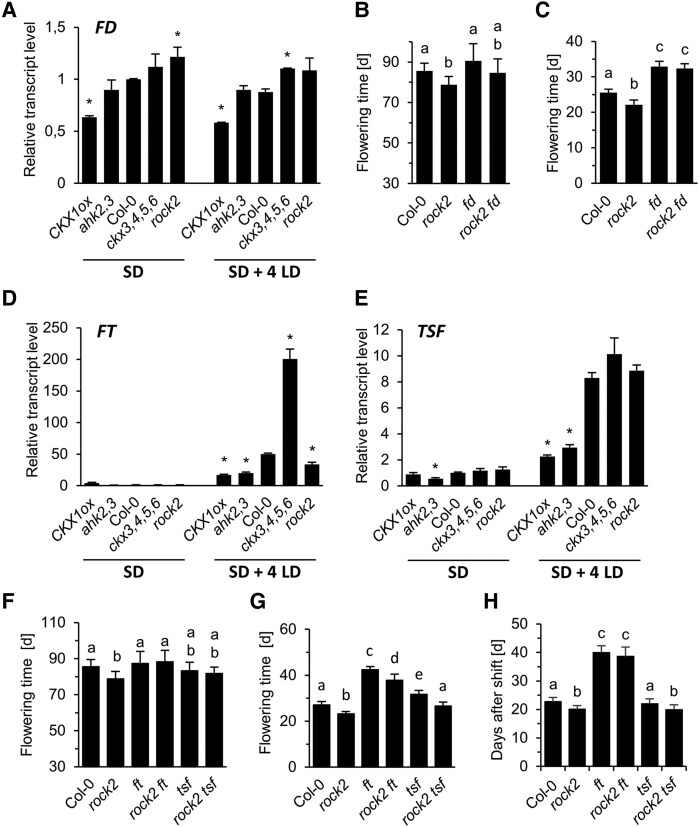
Analysis of the FT/TSF/FD functions in CK mutants. **A**, **D**, **E)** Expression of *FD* (A) in apices and of *FT* (D) and *TSF* (E) in leaves of plants grown for 4 wk under SD conditions and after floral induction by four additional LDs (SD + 4 LD). Expression levels were normalized to *PP2AA2*. The transcript levels in Col-0 were set to 1. Data are means ± SD (*n* = 3). Stars indicate statistically significant differences to Col-0 (one-way ANOVA (A) or Kruskal–Wallis **D, E)**, P ≤ 0.05). **B**, **C)**, Flowering time of *rock2*, *fd* and *rock2 fd* mutants grown under SD (B) or LD (C) conditions. Data presented are means± SD (*n* ≥ 15). d, days after sowing. Letters shared between the genotypes indicate no significant difference (Kruskal–Wallis, *P* < 0.05). F–H), Flowering time of *rock2 ft*, *rock2 tsf* and the respective single mutants grown under SD (F) or LD (G) conditions, or after a shift from SD to LD (H). Data presented are means ± SD (*n* = 15). Letters shared between the genotypes indicate no significant difference (Kruskal–Wallis (F) or one-way ANOVA **G, H)**, *P* < 0.05. followed by Tukey HSD (G and H), *P* < 0.05).

Next, we studied the function of the FD-FT/TSF module operating upstream of *SOC1* (Abe et al. 2005). The transcript levels of *FD*, encoding a central regulator of floral transition acting in the SAM with FT or TSF as partners, were reduced in apices of *CKX1ox* plants grown for 4 wk under SD conditions while *ckx3,4,5,6* and *rock2* plants had slightly higher *FD* mRNA levels (Fig. 5A). To analyze the function of FD in mediating CK signaling we introgressed the late flowering mutant *fd* (Abe et al. 2005; Wigge et al. 2005; Jaeger et al. 2013) into the *rock2* mutant. In SD, the *rock2 fd* double mutant showed an intermediate phenotype and flowered similar to its parental lines (Fig. 5B). On the other hand, under LD conditions *rock2 fd* flowered as late as *fd*, indicating that the promoting role of CK on the reproductive transition depends under permissive conditions on the presence of FD (Fig. 5C, Supplementary Fig. S3) and suggesting that the hormone feeds into the photoperiod pathway.

Transcript analysis of *FT* and *TSF* showed as expected only weak expression of these genes in leaves of plants grown under SD, but they were strongly induced in plants shifted to LD to induce flowering (Fig. 5, D and E). The shift to permissive conditions induced both genes also in CKX1ox but their transcripts reached only one-fourth to one-third of the levels reached in WT. Interestingly, the *ckx3,4,5,6* mutant showed a stronger induction of *FT* expression than in WT after the shift to LD, while transcript levels of *TSF* changed similar to WT (Fig. 5, D and E). These data show that CK is required for full induction of *FT* and *TSF* under permissive conditions.

The consequences of *ft* and *tsf* mutation on flowering time were analyzed in WT and *rock2* background. Under SD conditions, the *ft* and *tsf* mutants showed no changes of flowering time in the WT background, but, unexpectedly, *ft* prevented the flowering-promoting effect of *rock2* (Fig. 5F). When plants where grown continuously under LD, *rock2* caused earlier flowering in both late-flowering *ft* and *tsf* mutants suggesting either their functional redundancy or, alternatively, other factors supporting FD function (Fig. 5G). The functional relevance of FT was then tested further in a shift experiment. When plants were transferred from SD to LD conditions, FT was required to mediate rock2 function (Fig. 5H). Thus, surprisingly FT is relevant to mediate rock2 action under SD conditions, while its possible role under permanent LD is apparently masked.

### Components of the age pathway are involved in mediating CK action

We then assessed which of the flowering time pathways might be involved in mediating CK signaling to the central regulators. The age pathway was one option because it is important under SD conditions (Wang et al. 2009; Wu et al. 2009) and recent work has shown that CK regulates the juvenile-to-adult transition of leaves through the age pathway (Werner et al. 2021b). As the components of the age pathway regulating the juvenile-to-adult transition and flowering time under SD conditions are in part identical (Wang et al. 2009; Wu et al. 2009) it could be that CK uses age pathway components for both activities.

**Figure 6.**
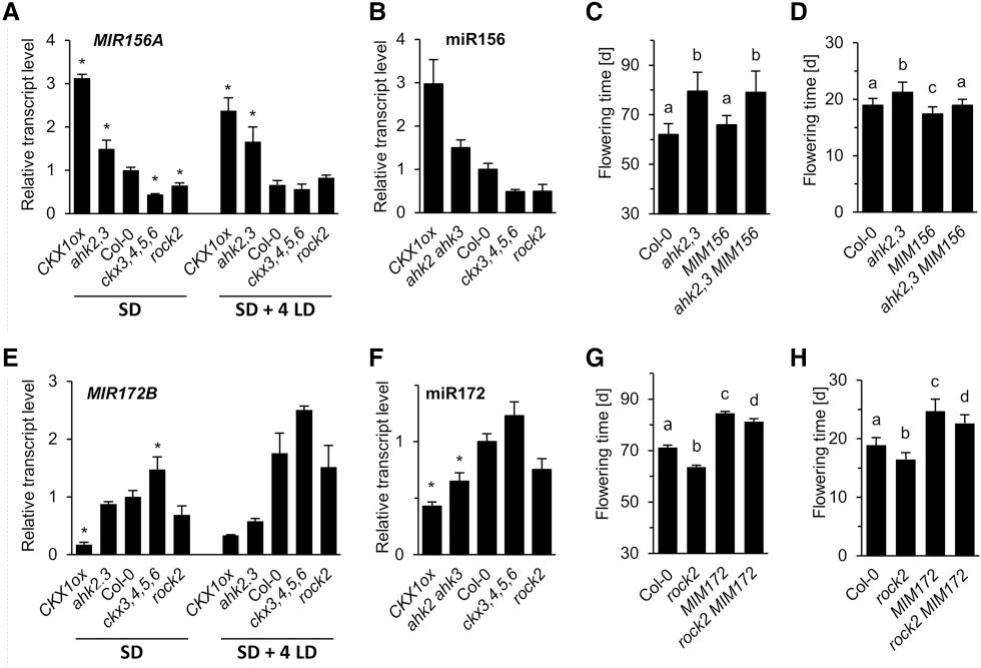
Analysis of miR156 and miR172 expression and function in CK mutants. **A) E)**, Relative abundance of *MIR156A* (A) and *MIR172B* (E) transcripts in apices of plants grown for 4 wk under SD conditions and after floral induction by four additional LDs (SD + 4 LDs). Expression levels were normalized to *PP2AA2*. The transcript levels in Col-0 were set to 1. Data are means ± SD (*n* = 3). **B) F)**, Relative abundance of miR156 (B) and miR172 (F) in plants grown for 4 wk under SD conditions. Total RNA was extracted from hand-dissected shoot apices. Expression levels were normalized to *TAFII15*. The transcript levels in Col-0 were set to 1. Data are means ± SD (*n* = 3). Statistical significance of differences to corresponding Col-0 was calculated by one-way ANOVA; * *P* < 0.05. **C,**  **D)**, **G, H)**, Flowering time of WT, *ahk2 ahk3 MIM156*  **C, D)** and *rock2 MIM172*  **G, H)** mutants and its progenitors grown under SD **C, G)** or LD **D, H)**. Data presented are means ± SD (*n* ≥ 15). Letters shared between the genotypes indicate no significant difference (Kruskal–Wallis; *P* < 0.05). d, days after sowing.

In shoot apices, transcript levels of *pri-miR156a* (*MIR156A*) and of the mature miR156 correlated negatively with the plant CK status (Fig. 6, A and B), which was observed over several weeks after germination (Supplementary Fig. S2B). The increase in *MIR156A* in CK-deficient plants was maintained also after a shift from SD to LD conditions (Fig. 6A). To explore the functional relevance of this negative correlation between miR156 and CK we introgressed the *35S:MIM156* (short: *MIM156*) gene, expressing a target mimic of miR156 (Franco-Zorrilla et al. 2007), into the late flowering *ahk2,3*. Blocking of miR156 had no effect on flowering time measured in days under SDs, neither in WT nor in the *ahk2,3* mutant (Fig. 6C). Under LD conditions, *MIM156* expression rescued the late flowering phenotype of the *ahk2,3* mutants (Fig. 6D), but only to the extent that it also accelerates flowering in WT. It appears that CK is acting during the transition to flowering principally downstream of miR156, similar to its action during vegetative phase change (VPC) (Werner et al. 2021b).

Expression analysis of *MIR172B* and miR172 revealed the opposite pattern than that of *MIR156A*/miR156. The levels of these transcripts were lower in apices of CK-deficient CKX1ox and elevated in *ckx3,4,5,6* mutants as compared to the WT at different developmental stages (Fig. 6, E and F, Supplementary Fig. S2C). *Rock2* was introgressed into *MIM172*, a miR172 mimic target (Todesco et al. 2010) causing late flowering under both SD and LD conditions (Fig. 6, G and H). The promoting activity of *rock2* on the floral transition was lowered to about one-third of its effect in WT in the *MIM172* background in both SD and LD (Fig. 6, G and H). The reduced effect of *rock2* in a background expressing *MIM172* indicates that *miR172* is at least partially involved in the earlier flowering of *rock2*.

### The role of transcription factors of the age pathway in mediating the CK flowering signal

**Figure 7.**
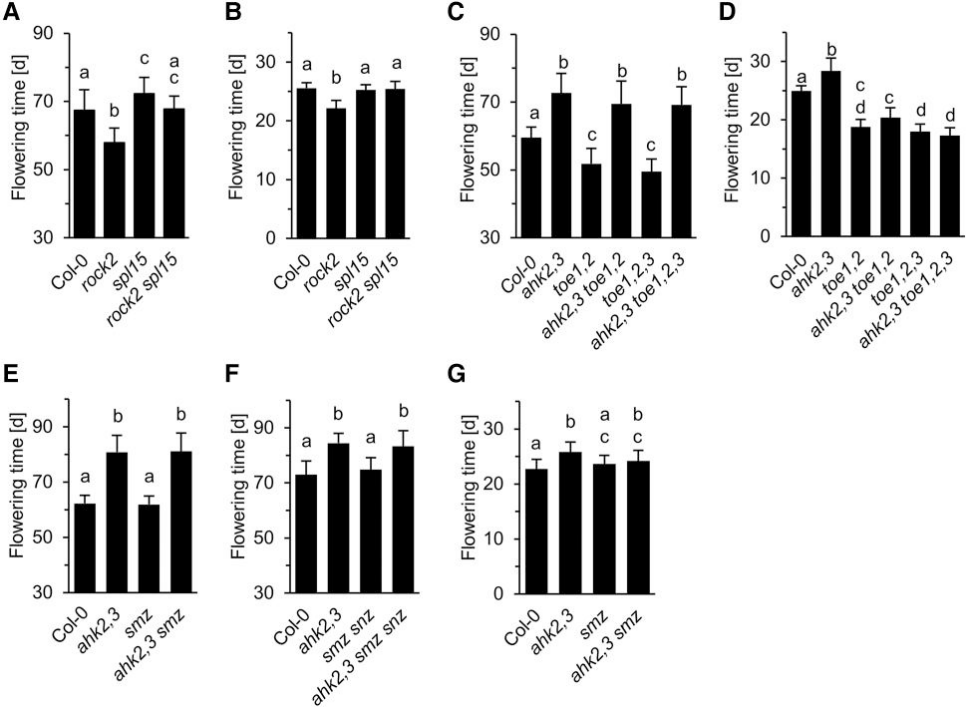
Functional analysis of miR156 and miR172 target genes. **A), B)** Flowering time of *rock2 spl15* and the respective single mutants grown under SD **A)** or LDs **B)**. *n* ≥ 27 **A)** and *n* ≥ 14 **B)**. **C), D)** Flowering time of *toe1*, *toe2*, and *toe3* mutant combinations grown under SD **C)** or LDs **D)**. *n* ≥ 22 **C)** and *n*  *=* 15 **D, E** to **G)** Flowering time of *smz* and *smz snz* mutants grown under SD **E, F)** or LDs **G)**. *n* ≥ 27 **E)**, *n* ≥ 21 **F)**, *n* ≥ 22 **G)**. All data are given as means ± SD. Letters shared between the genotypes indicate no significant difference (Kruskal–Wallis (A, **C, E, G)** or one-way ANOVA (B, **D, F)**, *P* < 0.05).

It was previously shown that the miR156-targeted gene *SPL15* regulates the transition to flowering under noninductive conditions and promotes expression of miR172 (Xu et al. 2016; Hyun et al. 2017). Therefore, we analyzed the potential relevance of SPL15 downstream of CK. Under SDs, the *rock2 spl15* mutant flowered significantly later than *rock2* (Fig. 7A), which suggests that SPL15 is needed for CK to become fully effective. Furthermore, the relative expression levels of *SPL15* are at different time points positively correlated with the CK content of different genotypes (Supplementary Fig. S2D). Interestingly, under inductive LDs lack of *SPL15* suppressed the early flowering phenotype of *rock2*, although and in agreement with Schwarz et al. (2008), the single *spl15* mutant did not show a flowering phenotype compared with the WT (Fig. 7B). This result reveals a latent function of SPL15 under LD conditions, which is needed by CK to cause earlier flowering.

Next, we explored the functional relevance of different miR172 target genes, which encode repressors of flowering, for the late flowering of *ahk2,3*. First we analyzed the *TOE* genes, which act downstream of CK in vegetative phase transition (Werner et al. 2021b). Under SDs the mutation of either two (*TOE1, TOE2*) or all three *TOE* genes did not alter the late flowering phenotype of *ahk2,3* mutants, whereas *TOE2* transcripts are clearly increased in CK-deficient plants (Fig. 7C, Supplementary Fig. S2E). This indicates that these floral repressors are not required for late flowering of CK-deficient plants or exhibit greater redundancy under SDs. In contrast, in plants grown under LD conditions mutation of *TOE* genes abrogated late flowering caused by impaired CK signal transduction (Fig. 7D). Thus, TOEs are required for late flowering of *ahk2,3* suggesting their functional involvement in the CK flowering pathway. A similar analysis was carried out with mutant lines that lack *SMZ* and its paralogue *SNZ*. Neither under SD nor under LD conditions these mutations influenced the late flowering phenotype of *ahk2 ahk3*, although *SMZ* is more strongly expressed in CK-deficient plants under SD conditions (Fig. 7E to G, Supplementary Fig. S2F).

## Discussion

This study provides genetic evidence that CK is a positive regulator of flowering time with greater relevance under SD than under LD conditions. The CK flowering pathway uses distinct CK signaling proteins, relies on central flowering time regulators and operates at least partly through components of the age pathway. Excitingly, the study has shown that the hormone acts as a root-derived signal to regulate the transition to flowering in the SAM.

### CK genes acting in the flowering pathway

**Figure 8.**
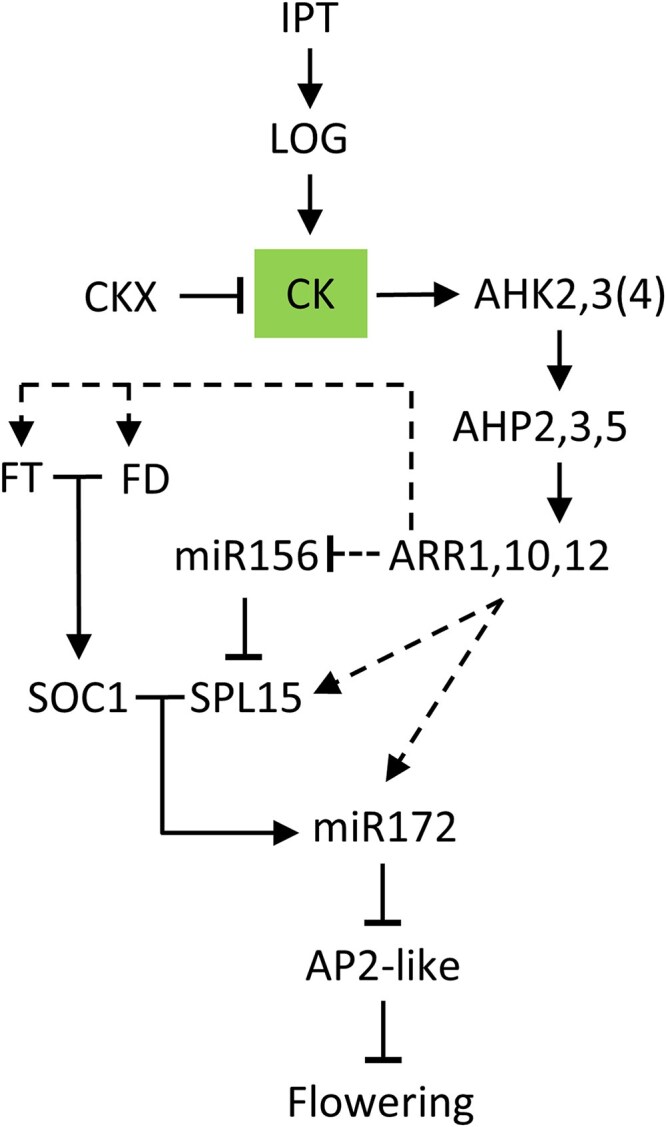
Model for the regulation of flowering time by CK. The model shows redundant activity of CK synthesizing and degrading enzymes. The CK signal is mediated through distinct receptors (AHKs), phosphotransmitters (AHPs) to B-type ARR transcription factors. Their links to components of known flowering pathways are based on transcript and genetic data and shown by dotted lines. CK, cytokinin.

Systematic analysis of CK metabolism and signaling mutants identified CK genes involved in regulating flowering time (Fig. 8). CK synthesis mutants having either a reduced concentration of iP- and *t*Z-type CKs (*ipt3,5,7*) or lacking *c*Z-type CKs (*ipt2,9*) (Miyawaki et al. 2006) flowered under SD 20 to 30 d later than WT (Table 1), indicating that iP-, *t*Z- and *c*Z-type CKs contribute to regulate flowering time. However, the *cypDM* mutants lacking root-derived *t*Z-type CK but having an increased concentration of iP-type CKs in the shoot (Kiba et al. 2013) flowered late. This suggests that iP-type CKs are less efficient (or even inefficient) in promoting flowering compared with *t*Z-type CKs. The role of *c*Z-type CK is interesting as these have generally lower biological activity than iP- and *t*Z-type CKs, and there is only limited knowledge about their functional relevance in *Arabidopsis thaliana* (Schäfer et al. 2015).

Overexpression of different *CKX* genes showed different degrees of impact on shoot growth and flowering time. Overexpression of *CKX1* or *CKX3* caused a strong reduction of shoot growth while shoot growth of *CKX2ox* and *CKX4ox* plants was only slightly affected (Werner et al. 2003; Supplementary Fig. S1). However, *CKX1ox*, *CKX3ox*, and *CKX4ox* showed strong retardation of flowering while *CKX2ox* plants showed a much weaker effect. These results revealed that the impact of CK on flowering time is not coupled to its effect on overall shoot growth and suggests that the different subcellular pools of CK metabolites degraded by individual CKX proteins (Werner et al. 2003; Galuszka et al. 2007; Köllmer et al. 2014; Niemann et al. 2015) differ in their relevance to regulate flowering time.

The CK flowering pathway uses distinct signaling components including all three receptors with a prevalent activity of AHK3 together with AHK2 or AHK4/CRE1, which are common combinations also for other CK activities in the shoot (Heyl et al. 2012). Consistent with their function in regulating flowering time, all three receptor genes are expressed in the SAM and in leaves (Higuchi et al. 2004; Nishimura et al. 2004; Mähönen et al. 2006) and active receptors are formed in these tissues (Stolz et al. 2011). Downstream of the receptors AHPs (AHP2, AHP3, and AHP5) and the RRBs ARR1, ARR10, and ARR12 mediate the effect of CK in regulating flowering time with a prevalent activity of ARR10. Notably, in rice a single RRB, EARLY HEADING DATE 1 (EHD1), is a regulator of flowering time (Doi et al. 2004; Cho et al. 2016) suggesting that the two-component signaling pathway regulates flowering in both dicot and monocot species.

### Root-derived CK controls flowering time

Our mutant analysis indicated that root-derived CK has a major role in regulating the transition to flowering in the SAM. Abolished export of *t*Z-type CK from roots in the *abcg14* mutant, lack of synthesis of the mainly root-derived *t*Z-type CK in the *cypDM* mutant or experimentally enhanced ectopic CK degradation in roots (*P10:CKX3*) delayed flowering time, particularly under SD (Table 1, Supplementary Table S1). Grafting experiments between WT and *abcg14* plants discriminated between root-derived and shoot-synthesized CK and demonstrated the functional relevance of root-derived CK in regulating flowering time. Under SD conditions root-derived CK appears to be a major long range flowering-promoting factor as its absence caused strong retardation of the transition to flowering (Fig. 3A). While wild-type root stocks completely rescued the late flowering of *abcg14* mutant scions, wild-type scions grafted onto *abcg14* rootstocks flowered as late as *abcg14* mutants. The influence of root-derived CK was less pronounced but still clearly visible under LD conditions, when the photoperiod pathway is predominant (Fig. 3B). These data unequivocally demonstrate a role of root-derived CK in regulating flowering time, which has been proposed previously but remained to be formally tested (Havelange et al. 2000; D'Aloia et al. 2011; Bernier 2013).

CK mediate environmental information to regulate developmental processes and may therefore fulfill a function as a systemic flowering time signal. Consistent with this hypothesis, previous reports suggested that the formation of CK in roots is induced by a leaf-derived sugar signal (Havelange et al. 2000; Bernier 2013). This is in line with the root upregulation of CK biosynthesis genes (*IPT3*, *IPT7*) in response to flowering-inducing stimuli such as longer photoperiods (Bouché et al. 2016). Similarly, *IPT3* and *CYP735A2* are induced in roots of *Arabidopsis* plants growing under elevated CO_2_ or in media with high sugar (Kiba et al. 2019). It is thus conceivable that sugar levels (or other yet unknown signals) inform roots about the nutritional status of aboveground organs, stimulate root CK biosynthesis which then translocates to the shoot apex via ABCG14 and promotes the transition to flowering. Future studies are needed to uncover the molecular events integrating CK activity with other pathways and unravel its ecological relevance. This could be related to the role of CK as a signal for nitrate availability acting as well as a flowering signal (Landrein et al. 2018; Olas et al. 2019).

### CK regulates plastochrone and flowering time by different pathways

There is generally a strong correlation between leaf number and transition to flowering and very often leaf number is recorded as a measure of flowering time (Koornneef et al. 1991). However, genetic studies comparing different *Arabidopsis* ecotypes had indicated that these traits are regulated by at least partially different pathways (Stratton 1998; Mendéz-Vigo et al. 2010; Salomé et al. 2011). A survey published by Pouteau et al. (2004) on early flowering mutants showed that in one-third of the 81 analyzed mutants, leaf number and flowering time were not coupled and both were differentially affected in different environmental and genetic context. In CK mutants the CK status clearly correlated with the time until bolting but not in all genotypes with leaf number at the bolting stage (Table 1). Both early and late flowering CK mutants having a higher or lower CK status may form less leaves until flowering, which might be explained by a bell-shaped dose-response curve of CK action (Ferreira and Kieber 2005). Gain-of-function alleles of the *AHK2* and *AHK3* receptor genes normalized both the longer plastochrone of *35S:CKX1* transgenic plants to the WT level, but only one of them (*rock2*) was able to reestablish flowering, which is consistent with distinct CK signaling pathways regulating these traits (Bartrina et al. 2017). Together, we conclude that CK regulates flowering time independent of leaf number confirming an earlier report by Bernier and Perilleux (2005). Interestingly, leaf number is a poor predictor of flowering time in plants with elevated or reduced levels of miR156 or SPL proteins (Poethig and Fouracre 2024) suggesting that the impact of CK on miR156 and/or *SPL* level may be causal for the decoupling of flowering time and leaf number in CK mutants.

### SOC1 and the FD-FT/TSF module are part of the CK flowering pathway

Our genetic analysis has confirmed that SOC1 as a central regulator of flowering is required for early flowering by CK under both LD and SD (Fig. 4) (D'Aloia et al. 2011). Interestingly, high transcript abundance of *SOC1* in genotypes with low CK was not sufficient to cause flowering. This could be due to the necessity for SOC1 to cooperate with SPL15 (Hyun et al. 2016), which is lower expressed in CK-deficient genotypes and thus could be rate-limiting. In addition, our analysis demonstrated the essential requirement of FD under LD, indicating that under permissive conditions CK feeds into the photoperiod pathway (Fig. 5C). The loss of FT delayed the early flowering of *rock2* under SD (Fig. 5F). This contrasts with D’Aloia et al. (2011) who found that under SD conditions TSF mediates the action of exogenous CK. FT was also required to respond rapidly to a shift from SD to LD (Fig. 5H) underpinning its role in mediating CK action and revealing a latent function for FT under SD. In contrast, neither FT nor TSF were needed for CK to be fully effective when plants were continuously grown under inductive conditions (Fig. 5G). This could be due to functional redundancy of FT and TSF or as yet unknown additional FD interaction partners including other proteins from the FT/TFL family (Kobayashi et al. 1999; Ryu et al. 2011).

### Age pathway components are involved in mediating the CK flowering signal

CK controls flowering time at least partially through the age pathway (Fig. 8). There is a remarkable impact of the plant’s CK status on the miR156 and miR172 transcript levels in SD-grown plants and after flower induction (Fig. 6; Supplementary Fig. S2, B and C). Low CK is associated with high *MIR156A*/miR156 and low *MIR172B*/miR172 abundance and high CK causes the inverse pattern, which confirms Werner et al. (2021b).

Recently, it was shown that miR156 has only a minor influence on the reproductive capacity (Zhao et al. 2023). However, SPL15, which has also been shown to be regulated independently of miR156 (Xu et al. 2021), promotes flowering in part via miR172 in both LD and SD, although its contribution is more important in SD (Hyun et al. 2016, 2017; O'Maoileidigh et al. 2021). SPL15 has been identified to be also an important transmitter of CK activity in promoting flowering under both SD and LD (Fig. 7, A and B). SPL15 additionally integrates age and GA flowering pathways at the SAM, where it cooperates with SOC1 to induce the expression of miR172 (Hyun et al. 2016). The functional relevance of the SPL15-SOC1 cooperation might be the cause for the ineffectivity of high *SOC1* expression in inducing the transition to flowering in CK-deficient genotypes (Fig. 4A) as *SPL15* expression is low (Supplementary Fig. S2D).

Because expression of a target mimicry of miR172 lowered the impact of CK on the floral transition (Fig. 6, G and H) it can be concluded that miR172 transmits at least part of the CK signal under both LD and SD. This could be realized by direct activation of *MIR172* genes by CK as is supported by the regulation of their expression by CK (Werner et al. 2021b) and the binding of RRBs to *MIR172* genes (Zubo et al. 2017; Xie et al. 2018). The gradual effect of *MIM172* on the flowering behavior of CK mutants suggests that additional factors are involved in the regulation of flowering time by CK. For example, SPL15 and SOC1 not only promote miR172 expression (Hyun et al. 2016) but both also regulate directly the target genes of miR172 suggesting that miR172 may be (partially) bypassed (Immink et al. 2012; Tao et al. 2012; Hyun et al. 2016).

We were unable to identify the target genes of miR172 for transducing the CK flowering signal under SD conditions, probably due to the high functional redundancy between these targets (Aukerman and Sakai 2003; Jung et al. 2007, 2011; Mathieu et al. 2009; Yant et al. 2010). On the other hand, under LD TOE transcription factors are clearly involved in mediating the consequences of reduced CK signaling (Fig. 7). Their inhibitory activity on flowering time takes place in leaves, where they inhibit *FT* expression (Mathieu et al. 2009; Zhang et al. 2015). It could thus be that at least part of the late flowering phenotype of CK-deficient mutants under LDs is realized in leaves, which is consistent with the promotive effect of CK on *FT/TSF* expression (Fig. 5, D and E). Noteworthy, expression of *TOE1* and *TOE2* as well as of *SMZ* was found to be rapidly downregulated by CK to less than half of their original level (Brenner et al. 2005) suggesting that in addition a direct regulation of these genes by CK may occur independent of miR172.

In sum, we discovered the involvement of age pathway components in CK regulation of flowering time and thus linking CK to miRNA activity. Recently, it has been reported that CK also regulates the juvenile-to-adult transition through age pathway components (Werner et al. 2021b). The 2 pathways show some similarities but also distinct differences and the age-dependent increase in reproductive competence is regulated independently of VPC (Zhao et al. 2023). However, root-derived CK plays a role in both the VPC and the transition to flowering, and the central CK signaling components are the same. In contrast, cZ-type CK affects only floral transition but not the juvenile-to-adult transition of leaves. Mutation of *AHP2*, *AHP3*, and *AHP5* had a strong impact on flowering but not on VPC (Werner et al. 2021b). Other differences concern the relative importance of some age pathway components. SPL15 is important for flowering time control by CK while in VPC the activity of SPLs downstream of CK appears to be highly redundant. miR172 has an important role in both processes, but while TOE1 and TOE2 are the main targets in VPC (Werner et al. 2021b) the role of AP2-like transcription factors is more diverse in the CK flowering pathway.

Altogether these findings integrate the activity of CK in the complex and dynamic scenario of regulation of the floral transition, in which the hormone contributes to its plasticity in particular under nonpermissive conditions, and set a framework for future investigations into its specific role in the highly connected flowering pathways.

## Materials and methods

### Plant material and growth conditions

The Columbia (Col-0) ecotype *of Arabidopsis thaliana* was used as the WT and all mutants and transgenic plants listed in Supplementary Table S2 were in Col-0 background. The reduced or enhanced CK content of CK metabolism and transport mutants used in this study has been published, including for *log3,4,7* by Kuroha et al. (2009); *ipt2,9* and *ipt3,5,7* by Miyawaki et al. (2006); *cypDM* by Kiba et al. (2013); *ckx3,5* by Bartrina et al. (2011); *abcg14* by Zhang et al. (2014) and Ko et al. (2014); and for *CKXox* plants by Werner et al. (2003) and Nishiyama et al. (2011). An increase in CK concentration for *ahk2,3* has been reported by Riefler et al. (2006) and the decreased CK concentration in *rock2* plants by Bartrina et al. (2017). Multiple mutants were generated by genetic crossings and verified by genotyping. The *ckx7-1* mutant was a previously uncharacterized knock-out allele from the GABI-KAT collection (Line ID: 363C02) (Rosso et al. 2003), it is described in Supplementary Fig. S4. Oligonucleotides used for genotyping are listed in Supplementary Table S3. All plants were grown on soil under LD (16 h of light/8 h of darkness) or SD conditions (8 h of light/16 h of darkness) at 22 °C.

### Flowering time measurement

Flowering time was scored as days after sowing and as number of rosette leaves at bolting when the bolting shoot was about 0.5 cm long. All flowering time measurements were repeated at least twice with similar results. In order to synchronize germination, seeds were placed in the cold (2 d at 4 °C).

### Micrografting

Grafting was performed in asceptically grown 5-d-old seedlings (in LD conditions) or 6-d-old seedlings (in SD conditions) as described in Schulze et al. (2019). Graft formation was evaluated 8 d after the procedure and seedlings were transferred to soil 13–14 d after grafting ensuring the absence of adventitious roots. Flowering time was scored as described above. Grafting experiments were performed at the Leibniz Institute of Plant Biochemistry (IPB) in Halle with plant growth conditions as close as possible to those used for all other experiments at FU Berlin.

### RNA extraction and analysis of gene expression

Total RNA was extracted from hand-dissected shoot apices, leaves or whole rosettes as indicated at Zeitgeber 4 (4 h after the beginning of the light period) with the TRIzol method according to the manufacturer (Thermo Fisher Scientific). For cDNA synthesis, 1 *µ*g of total RNA was used in a 10 *µ*L SuperScript III Reverse Transcriptase reaction (Thermo Fisher Scientific). First strand complementary DNA synthesis was primed with a combination of oligo (dT) primers and random hexamers. Real-time PCR using FAST SYBR Green I technology was performed on an Applied Biosystems 7500 Real-Time PCR System (Thermo Fisher) or CFX96 Touch Real-Time PCR Detection System (Bio-Rad). Relative transcript abundance of each gene was calculated based on the ΔΔCt method (Livak and Schmittgen 2001). The gene *PROTEIN PHOSPHATASE 2A SUBUNIT A2* (*PP2AA2*) was used for normalization. Semi-quantitative measurement of *CKX7* expression (Supplementary Fig. S4) was performed as described in Bartrina et al. (2011) using the *ACTIN2* gene as expression control. Primers used for reference genes and genes of interest are listed in Supplementary Table S4.

### Stem-loop RT-qPCR

For detection of mature miRNAs, mix 1 contained 500 ng of total RNA, 1 mm of dNTP mix, 25 nm TAFII15-StLp-cDNA_rv as an internal control for qRT analyses, and 25 nm of the respective miRNA-specific stem-loop primer (Supplementary Table S4). Stem-loop RT primers were designed according to Chen et al. (2005). Following the addition of mix 2 (first strand buffer, 4 mm DTT, 0.6 U/µL RNaseOUT (ThermoFisher), SuperScript III) resulting in a total volume of 12.5 µL, samples were incubated for 30 min at 16 °C, 30 min at 50 °C and 15 min at 70 °C. Undiluted cDNA was used in the RT-qPCRs. For RT-qPCR analyses, *TBP-ASSOCIATED FACTOR II 15 (TAFII15)* served as reference genes. All RT-qPCR primers used in this study are listed in Supplementary Table S4. RT-qPCR was performed with the CFX96 Real-Time Touch System (Bio-Rad) using SYBR Green I as DNA-binding dye. Gene expression data analyses were carried out according to Vandesompele et al. (2002).

### Statistical analyses

Student’s *t*-test, one-way ANOVA or Kruskal–Wallis test as specified in the figure legends coupled to a multiple comparison test were used to determine the statistical significance of flowering time differences.

### Accession numbers

Sequence data for the genes described in this article can be found in the GenBank/EMBL databases under the following accession numbers: *ABCG14* (AT1G31770); *AHK2* (AT5G35750); *AHK3* (AT1G27320); *AHK4* (AT2G01830); *AHP2* (AT3G29350); *AHP3* (AT5G39340); *AHP5* (AT1G03430); *AP2* (AT4G36920); *ARR3* (AT1G59940); *ARR4* (AT1G10470); *ARR5* (AT3G48100); *ARR6* (AT5G62920); *ARR8* (AT2G41310); *ARR9* (AT3G57040); *ARR1* (AT3G16857); *ARR2* (At4G16110); *ARR10* (At4G31920); *ARR11* (AT1G67710); *ARR12* (AT2G25180); *CKX1* (AT2G41510); *CKX2* (AT2G19500); *CKX3* (AT5G56970); *CKX4* (AT4G29740); *CKX5* (AT1G75450); *CKX6* (AT3G63440); *CKX7* (At5G21482); *CYP735A1* (AT5G38450); *CYP735A2* (AT1G67110); *FD* (AT4G35900); *FT* (AT1G65480); *IPT2* (AT2G27760); *IPT3* (AT3G63110); *IPT5* (AT5G19040); *IPT7* (AT3G23630); *IPT9* (AT5G20040); *LOG3* (AT2G37210); *LOG4* (AT3G53450); *LOG7* (AT5G06300); *MIR156A* (AT2G25095); *MIR172B* (AT5G04275); *PP2AA2* (AT3G25800); *SMZ* (AT3G54990); *SNZ* (AT2G39250); *SOC1* (AT2G45660); *SPL15* (AT3G57920); *TAFII15* (AT4G31720); *TOE1 (AT2G28550); TOE2* (AT5G60120); *TOE3* (AT5G67180); *TSF* (AT4G20370).

## Supplementary Material

kiaf204_Supplementary_Data

## Data Availability

The data underlying this article are available in the article and in its online supplementary material.

## Supplementary data

The following materials are available in the online version of this article.

**Supplementary Figure S1.** Photographs of CK mutants and transgenic lines.

**Supplementary Figure S2.** Expression of *SOC1* and age pathway genes in the shoot at different developmental stages.

**Supplementary Figure S3.** Late flowering phenotype of *rock2 fd* under LD conditions.

**Supplementary Figure S4.** Characterization of the *ckx7-1* T-DNA insertion allele.

**Supplementary Table S1.** Flowering time of Arabidopsis CK metabolism and signaling mutants grown under LD-conditions.

**Supplementary Table S2.** Mutants and transgenic lines used in this study.

**Supplementary Table S3.** Oligonucleotides used for genotyping.

**Supplementary Table S4.** Oligonucleotides used for gene expression analyses.

## Dive Curated Terms

The following phenotypic, genotypic, and functional terms are of significance to the work described in this paper:

AP2 Gramene: AT4G36920
AP2 Araport: AT4G36920

CYP735A2 Gramene: AT1G67110
CYP735A2 Araport: AT1G67110

MIR172D Gramene: AT3G55512
MIR172D Araport: AT3G55512

MIR172E Gramene: AT5G59505
MIR172E Araport: AT5G59505

PYK10 Gramene: AT3G09260
PYK10 Araport: AT3G09260

AHK2 Gramene: AT5G35750
AHK2 Araport: AT5G35750

TSF Gramene: AT4G20370
TSF Araport: AT4G20370

CYP735A1 Gramene: AT5G38450
CYP735A1 Araport: AT5G38450

AHK3 Gramene: AT1G27320
AHK3 Araport: AT1G27320

SMZ Gramene: AT3G54990
SMZ Araport: AT3G54990

TOE2 Gramene: AT5G60120
TOE2 Araport: AT5G60120

PCR Gramene: Polymerase Chain Reaction
PCR Araport: Polymerase Chain Reaction

TOE1 Gramene: At2g28550
TOE1 Araport: At2g28550

MIR156A Gramene: AT2G25095
MIR156A Araport: AT2G25095

PP2AA2 Gramene: AT3G25800
PP2AA2 Araport: AT3G25800

SPL15 Gramene: AT3G57920
SPL15 Araport: AT3G57920

MIR172B Gramene: AT5G04275
MIR172B Araport: AT5G04275

TOE3 Gramene: AT5G67180
TOE3 Araport: AT5G67180

MIR172A Gramene: AT2G28056
MIR172A Araport: AT2G28056

FD Gramene: AT4G35900
FD Araport: AT4G35900
