# Supplementary material for: Root-derived cytokinin regulates Arabidopsis flowering time through components of the age pathway
Source: Plant Physiol. 2025 Jun 25;198(3):kiaf204. doi: 10.1093/plphys/kiaf204 (PMC12311289; doi:10.1093/plphys/kiaf204)
Supplement: kiaf204_Supplementary_Data [file kiaf204_supplementary_data.zip › Bartrina et al_Supplemental figures.pdf]

Root-derived cytokinin regulates *Arabidopsis thaliana* flowering time through components of the age pathway

Supplemental material

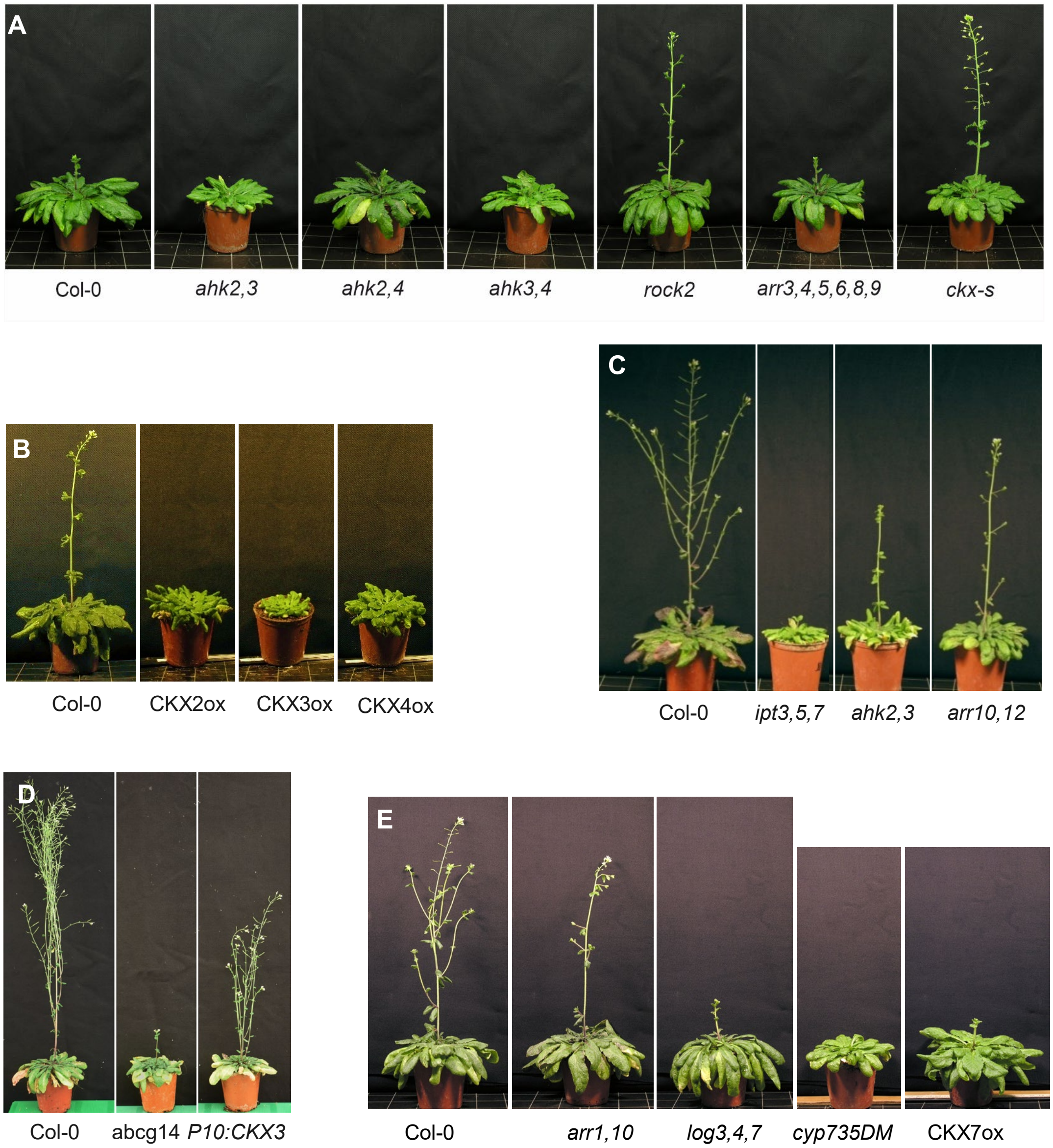

Supplemental Figure S1 Photographs of cytokinin mutants. Wild type (Col-0) and mutants grown (A) 74 d, (B) 88 d, (C) 91 d, (D) 110 d, and (E) 79 d under short-day conditions. The diameter of the pots is 6 cm.

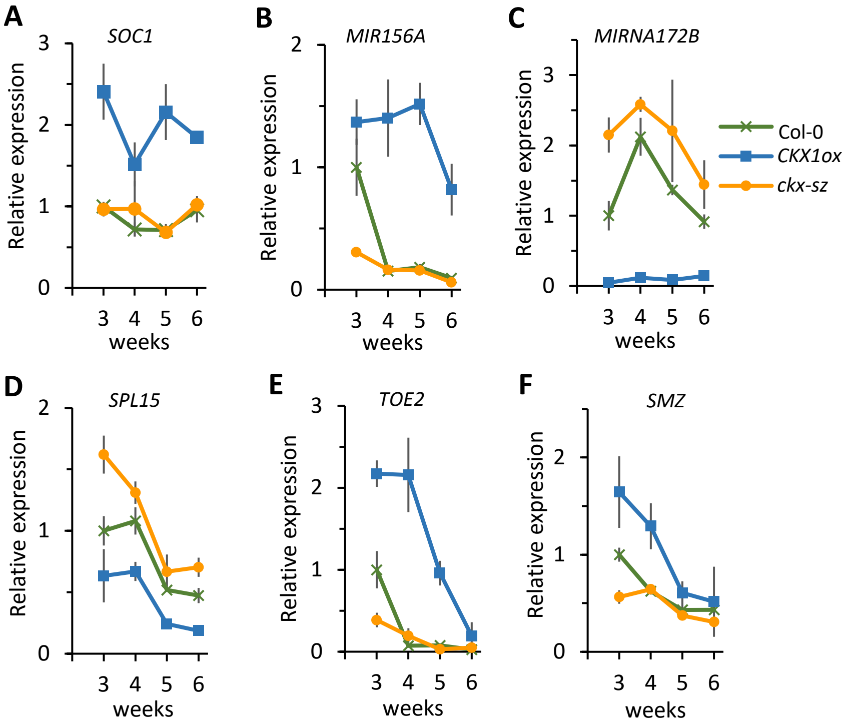

Supplemental Figure S2 Expression of *SOC1* and age pathway genes in the shoot at different developmental stages. (A–F) Graphs depict the relative expression of genes in shoots of short-day grown plants three to six weeks after germination as analyzed by qRT-PCR. The expression of samples from three-week-old Col-0 was set to 1. Error bars indicate SEM of three independent biological replicates.

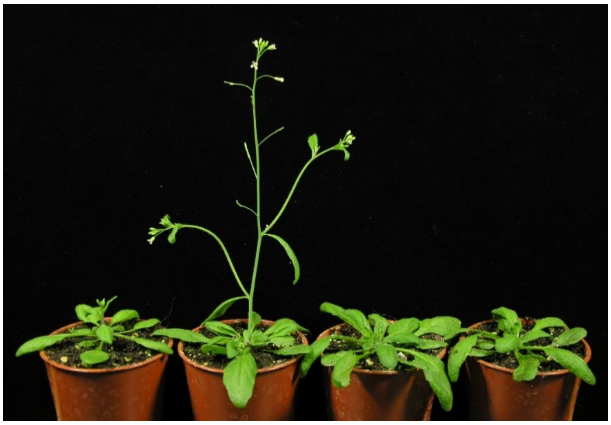

Supplemental Figure S3 Late flowering phenotype of *rock2 fd* under long day. Wild type, *rock2*, *fd* and *rock2 fd* double mutant grown 26 days under long-day conditions. The diameter of the pots is 6 cm. This figure supports Figure 5C.

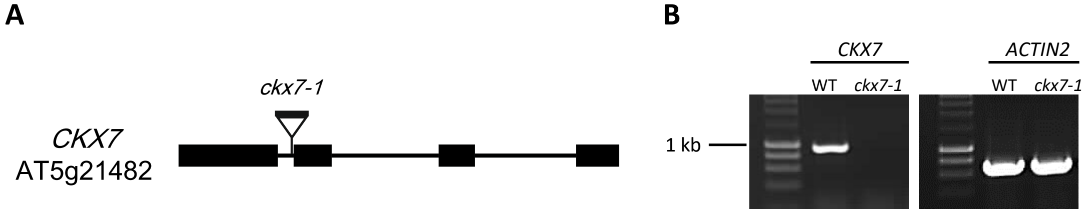

Supplemental Figure S4 Characterization of the *CKX7* T-DNA insertion allele. A, Position of T-DNA insertion in the *ckx7-1* mutant allele. The insertional mutant was identified by PCR screening and the site of insertion determined by DNA sequencing of the border fragments. Black boxes represent exons, lines indicate introns and the triangle indicates the T-DNA insertion site. B, Expression of *CKX7* in wild type (WT) and in the insertional mutant. RNA from 10-d-old seedlings was used as template for the RT-PCR. *ACTIN2* was used as an expression control. The figure describes a mutant used to generate *ckx-s* shown in Figure 1.
