# Supplementary material for: Root-derived cytokinin regulates Arabidopsis flowering time through components of the age pathway
Source: Plant Physiol. 2025 Jun 25;198(3):kiaf204. doi: 10.1093/plphys/kiaf204 (PMC12311289; doi:10.1093/plphys/kiaf204)
Supplement: kiaf204_Supplementary_Data [file kiaf204_supplementary_data.zip › Bartrina et al_Supplemental tables.pdf]

Root-derived cytokinin regulates *Arabidopsis thaliana* flowering time through components of the age pathway

Supplemental material

Supplemental Table S1 Flowering time of *Arabidopsis* cytokinin metabolism and signaling mutants grown in long days.

Genotype	Days	*n*
**Experiment 1 – Metabolism/ Signaling**		
Col-0	21.0 ± 1.1	29
ckx3,4,5,6	**18.7** ± 0.6	29
CKX1ox	**32.2** ± 2.5	15
rock2	**19.4** ± 0.9	29
**Experiment 2 – Metabolism**		
Col-0	25.0 ± 1.3	21
ckx-s	**21.7** ± 1.1	42
**Experiment 3 - Metabolism**		
Col-0	19.2 ± 1.4	28
cypDM	**21.3** ± 1.7	28
P10:CKX3	**21.2** ± 1.1	28
**Experiment 4 - Transport**		
Col-0	22.0 ± 1.8	28
abcg14	**26.6** ± 4.0	27
**Experiment 5 - Signaling**		
Col-0	22.2 ± 1.7	26
ahk2 ahk3	**25.0** ± 2.6	26
ahk2 ahk4	**24.3** ± 1.9	26
ahk3 ahk4	22.4 ± 2.2	26
**Experiment 6 - Signaling**		
Col-0	23.5 ± 2.0	28
ahp2,3,5	**33.3** ± 3.1	28
**Experiment 7 - Signaling**		
Col-0	27.5 ± 2.0	26
arr1 arr10	27.3 ± 2.0	28
arr1 arr12	26.4 ± 1.2	27
arr10 arr12	**29.6** ± 2.5	26
arr1,10,12	**45.3** ± 5.6	23

Flowering time is given as days after sowing. Genotypes written in red/blue indicates their increased/decreased cytokinin content or signaling. Data presented are means ± SD. Numbers in bold indicate significant differences ($P < 0.05$) compared to wild type as calculated by Student´s *t*-test (experiment 3) and one-way ANOVA (all other experiments). n.a., not analyzed.

Supplemental Table S2. Mutants and transgenic lines used in this study.

**Genotype**	**Named in this**	**Reference**
rock2	*rock2*	Bartrina et al., 2017
ahk2-2tk ahk3-3	*ahk2 ahk3*	Higuchi et al., 2004
ahk2-2tk cre1-12	*ahk2 ahk4*	Higuchi et al., 2004
ahk3-3 cre1-12	*ahk3 ahk4*	Higuchi et al., 2004
arr1-3	*arr1*	Mason et al., 2005
arr10-5	*arr10*	Argyros et al., 2008
arr12-1	*arr12*	Mason et al., 2005
arr1-3 arr2-GK	*arr1 arr2*	this work
arr1-3 arr10-5	*arr1 arr10*	Argyros et al., 2008
arr1-3 arr12-1	*arr1 arr12*	Mason et al., 2005
arr2-GK arr10-5	*arr2 arr10*	Frank et al., 2020
arr2-GK arr12-1	*arr2 arr12*	Frank et al., 2020
arr10-5 arr12-1	*arr10 arr12*	Argyros et al., 2008
arr1-3 arr2-GK arr11-3	*arr1,2,11*	Werner et al., 2021
arr1-3 arr2-GK arr10-5	*arr1,2,10*	Werner et al., 2021
arr1-3 arr2-GK arr12-1	*arr1,2,12*	Werner et al., 2021
arr1-3 arr10-5 arr11-3	*arr1,10,11*	Werner et al., 2021
arr1-3 arr10-5 arr12-1	*arr1,10,12*	Mason et al., 2005
arr1-3 arr11-3 arr12-1	*arr1,11,12*	Werner et al., 2021
arr2-GK arr10-5 arr11-3	*arr2,10,11*	Werner et al., 2021
arr2-GK arr10-5 arr12-1	*arr2,10,12*	Frank et al., 2020
arr2-GK arr11-3 arr12-1	*arr2,11,12*	Werner et al., 2021
arr10-5 arr11-3 arr12-1	*arr10,11,12*	this work
arr1-3 arr2-GK arr10-5 arr11-3	*arr1,2,10,11*	this work
arr2-GK arr10-5 arr11-3 arr12-1	*arr2,10,11,12*	this work
arr3,4,5,6,8,9	*arr3,4,5,6,8,9*	To et al., 2004
35S:CKX1	*CKX1ox*	Werner et al., 2003
35S:CKX2	*CKX2ox*	Werner et al., 2003
35S:CKX3	*CKX3ox*	Werner et al., 2003
35S:CKX4	*CKX4ox*	Werner et al., 2003
35S:CKX7	*CKX7ox*	Köllmer et al., 2014
ckx7-1	*ckx7*	this work
ckx2-1 ckx3-1	*ckx2 ckx3*	Bartrina et al., 2011
ckx3-1 ckx6-2	*ckx3 ckx6*	Bartrina et al., 2011
ckx3-1 ckx7-1	*ckx3 ckx7*	this work
ckx3-1 ckx4-1 ckx5-1 ckx6-2	*ckx3,4,5,6*	Werner et al., 2021
ckx2-1 ckx3-1 ckx4-1 ckx5-1 ckx6-2	*ckx-s*	this work
cyp735a1-2 cyp735a2-2	*cypDM*	Kiba et al., 2013
ipt2-1 ipt9-1	*ipt2 ipt9*	Miyawaki et al., 2006
ipt3-2 ipt5-2 ipt7-1	*ipt3,5,7*	Miyawaki et al., 2006
log3-1 log4-3 log7-1	*log3,4,7*	Kuroha et al., 2009
pPYK10:CKX3	*pPYK10:CKX3*	Werner et al., 2010
ahp2 ahp3 ahp5-2	*ahp2,3,5*	Hutchison et al., 2006
abcg14-2	*abcg14*	Zhang et al., 2014
ft-10	*ft*	Yoo et al., 2005
tsf-1	*tsf*	Yamaguchi et al., 2005

ft-10 tsf-1	*ft tsf*	Jang et al., 2009
fd-3	*fd*	NASC (N678498)
soc1-2	*soc1*	Lee et al., 2000
p35S:MIM156	*MIM156*	plasmid from NASC (N9953)
p35S:MIM172	*MIM172*	plasmid from NASC (N783241)
spl15-1	*spl15*	Schwarz et al., 2008 NASC (N67867)
smz-4	*smz*	this work (NASC N664087)
snz-1	*snz*	Mathieu et al., 2009
toe1-2 toe2-1	*toe1 toe2*	Aukerman and Sakai, 2003
toe3-2	*toe3*	Jung et al., 2014

Supplemental Table S3. Oligonucleotides used for genotyping.

Allele	Sequence (5'- 3')	
ckx7-1	CGGAAAATCTACGGATGGTG<br>TTAGCCGTCCGATCAATCTC<br><br>ATATTGACCATCATACTCATTGC<br>TTAGCCGTCCGATCAATCTC	 <br><br>LB GABI
ft-10	TTTTCCACCAACTTCTTGCAT<br>AATTTAGCTTGGGTGTGGGC<br><br>ATATTGACCATCATACTCATTGC<br>AATTTAGCTTGGGTGTGGGC	 <br><br>LB GABI
tsf-1	CAACCCTCACCAACGAGAAT<br>TTTCCAGTGGTGGCAGGTAT<br><br>ATTTTGCCGATTTCGGAAC<br>TTTCCAGTGGTGGCAGGTAT	 <br><br>LBb1.3-SALK
fd-3	TCCCTCTCTGCGTGTAGGAT<br>AGGAGGTGGAAAAGGAGAGC<br><br>ATTTTGCCGATTTCGGAAC<br>AGGAGGTGGAAAAGGAGAGC	 <br><br>LBb1.3-SALK
soc1-2	GCAGAGAGAGAAGAGACGAGT<br>CCACAAAAGGCCAATCAAAT<br><br>ATTTTGCCGATTTCGGAAC<br>CCACAAAAGGCCAATCAAAT	 <br><br>LBb1.3-SALK
spl15-1	Wang et al., 2009	
smz-4	GTGGTGGCTGATGCTCGTC<br>AATCATCCACGACGAAATTGATGTCTG<br><br>TTTGATTTGTAGATCTTCTCTGACAAC<br>ATTTTGCCGATTTCGGAAC	 <br><br><br>LBb1.3-SALK
snz-1	AGGTCCCCAACACGTTCCATT<br>ATCCAACCACTCATTTCCGGG<br><br>ATTTTGCCGATTTCGGAAC<br>ATCCAACCACTCATTTCCGGG	 <br><br>LBb1.3-SALK
toe1-2	GAAGAGTTTGTGCATATACTGCG<br>GAAGGGAAGTGAAAGAGCCTC<br><br>ATTTTGCCGATTTCGGAAC<br>GAAGGGAAGTGAAAGAGCCTC	 <br><br>LBb1.3-SALK
toe2-1	AGTTGTGCTCTACACGAACGG<br>TCCCAGCAGAAATCAGTTCAC<br><br>AGTTGTGCTCTACACGAACGG<br>ATTTTGCCGATTTCGGAAC	 <br><br><br>LBb1.3-SALK
toe3-2	ATCCTGCACCTCTCTAATGATG<br>GACACTATTGAAACCGGACCA<br><br>GCCTTTTCAGAAATGGATAAATAGCCTTGCTTCC<br>GACACTATTGAAACCGGACCA	 <br><br><br>LB1-SAIL
rock2	Bartrina et al., 2017	

ahk2-2tk	Higuchi et al., 2004
ahk3-3	Higuchi et al., 2004

Supplemental Table S4. Oligonucleotides used for qRT-PCR.

Gene	Sequence (5’- 3’)
FT	GCTACAACTGGAACAACCTTTGGC<br>TGAATTCCTGCAGTGGGACTTGG
TSF	AGTAAGAGGCAGCCACAGGA<br>CAATGAGGTGGTGTGCTACG
FD	CCGCGCTAGGAAACAGGCTTATAC<br>TCTGCCTGCAAGTGAGCAACTTC
SOC1	TTCGCCAGCTCCAATATGCAAG<br>TGCTGACTCGATCCTTAGTATGCC
MIR156A	TGGGACAAGAGAAACGCAAAG<br>TGAGCACGCAAGAGAAGCAAGT
MIR172B	GTTGTTTGTAGGCGCAGCAC<br>GCAGCATCATCAAGATTCTCATATAC
PP2AA2	CGTGCGGTGTCTCTTCTT<br>TTTGATGTTTGGAACTCTGTCTTT
TAFII15	GAATCACGGCCAACAATC<br>ACTCTTAGCCAAGTAGTGCTCC

Primers used for stem-loop cDNA synthesis

TAFII15-StLp-cDNA_rv	CTATTCGTCCCTTGTTG
miR156/miR157-StLp	GTCGTATCCAGTGCAGGGTCCGAGGTATTCGCACTGGATACGACGTGC<br>T
miR172abe-StLp	GTCGTATCCAGTGCAGGGTCCGAGGTATTCGCACTGGATACGACATGC<br>A

qRT primers used for detection of mature miRNAs

universal reverse primer	CAGTGCAGGGTCCGAGGT
miRNA156 primer	CCGTGGTGACAGAAGAGAGTGA
miRNA172 primer	GTCCGTGGAGAATCTTGATGATG

Argyros RD, Mathews DE, Chiang YH, Palmer CM, Thibault DM, Etheridge N, Argyros DA, Mason MG, Kieber JJ, Schaller GE (2008) Type B response regulators of *Arabidopsis* play key roles in cytokinin signaling and plant development. Plant Cell **20:** 2102-2116

Aukerman MJ, Sakai H (2003) Regulation of flowering time and floral organ identity by a microRNA and its *APETALA2*-like target genes. Plant Cell **15:** 2730-2741

Bartrina I, Otto E, Strnad M, Werner, T Schmülling T (2011) Cytokinin regulates the activity of reproductive meristems, flower organ size, ovule formation, and thus seed yield in *Arabidopsis thaliana*. Plant Cell **23:** 69-80

Bartrina I, Jensen H, Novák O, Strnad M, Werner T, Schmülling T (2017) Gain-of-function mutants of the cytokinin receptors AHK2 and AHK3 regulate plant organ size, flowering time and plant longevity. Plant Physiol **173:** 1783–1797

Frank M, Cortleven A, Novák O, Schmülling T (2020) Root-derived *trans*-zeatin cytokinin protects *Arabidopsis* plants against photoperiod stress. Plant Cell Environ **43:** 2637-2649

Higuchi M, Pischke MS, Mähönen AP, Miyawaki K, Hashimoto Y, Seki M, Kobayashi M, Shinozaki K, Kato T, Tabata S, Helariutta Y, Sussman MR, Kakimoto T (2004) In planta functions of the Arabidopsis cytokinin receptor family. Proc Natl Acad Sci USA **101:** 8821-8826

Hutchison CE, Li J, Argueso C, Gonzalez M, Lee E, Lewis MW, Maxwell BB, Perdue TD, Schaller GE, Alonso JM, Ecker JR, Kieber JJ (2006) The Arabidopsis histidine phosphotransfer proteins are redundant positive regulators of cytokinin signaling. Plant Cell **18:** 3073-3087

Jang S, Torti S, Coupland G (2009) Genetic and spatial interactions between FT, TSF and SVP during the early stages of floral induction in *Arabidopsis*. Plant J **60:** 614-625

Jung JH, Lee S, Yun J, Lee M, Park CM (2014) The miR172 target *TOE3* represses *AGAMOUS* expression during *Arabidopsis* floral patterning. Plant Sci **215-216:** 29-38

Kiba T, Takei K, Kojima M, Sakakibara H (2013) Side-chain modification of cytokinins controls shoot growth in *Arabidopsis.* Dev Cell **27:** 452-461

Köllmer I, Novák O, Strnad M, Schmülling T, Werner T (2014) Overexpression of the cytosolic cytokinin oxidase/dehydrogenase (CKX7) from *Arabidopsis* causes specific changes in root growth and xylem differentiation. Plant J **78:** 359-371

Kuroha T, Tokunaga H, Kojima M, Ueda N, Ishida T, Nagawa S, Fukuda H, Sugimoto K, Sakakibara H (2009) Functional analyses of *LONELY GUY* cytokinin-activating enzymes reveal the importance of the direct activation pathway in *Arabidopsis*. Plant Cell **21:** 3152-3169

Lee H, Suh SS, Park E, Cho E, Ahn JH, Kim SG, Lee JS, Kwon YM, Lee I (2000) The AGAMOUS-LIKE 20 MADS domain protein integrates floral inductive pathways in *Arabidopsis*. Genes Dev **14:** 2366-2376

Mason MG, Mathews DE, Argyros DA, Maxwell BB, Kieber JJ, Alonso JM, Ecker JR, Schaller GE (2005) Multiple type-B response regulators mediate cytokinin signal transduction in *Arabidopsis*. Plant Cell **17:** 3007-3018

Mathieu J, Yant LJ, Murdter F, Kuttner F, Schmid M (2009) Repression of Flowering by the miR172 Target SMZ. PLoS Biol **7:** e1000148

Miyawaki K, Tarkowski P, Matsumoto-Kitano M, Kato T, Sato S, Tarkowska D, Tabata S, Sandberg G, Kakimoto T (2006) Roles of Arabidopsis ATP/ADP isopentenyltransferases and tRNA isopentenyltransferases in cytokinin biosynthesis. Proc Natl Acad Sci USA **103:** 16598-16603

Schwarz S, Grande AV, Bujdoso N, Saedler H, Huijser P (2008) The microRNA regulated SBP-box genes *SPL9* and *SPL15* control shoot maturation in *Arabidopsis*. Plant Mol Biol **67:** 183-195

To JPC, Haberer G, Ferreira FJ, Deruere J, Mason MG, Schaller GE, Alonso JM, Ecker JR, Kieber JJ (2004) Type-A *Arabidopsis* response regulators are partially redundant negative regulators of cytokinin signaling. Plant Cell **16:** 658-671

Wang JW, Czech B, Weigel D (2009) miR156-regulated SPL transcription factors define an endogenous flowering pathway in *Arabidopsis thaliana*. Cell **138:** 738-749

Werner S, Bartrina I, Schmülling T (2021) Cytokinin regulates vegetative phase change in *Arabidopsis thaliana* through the miR172/TOE1-TOE2 module. Nat Commun **12:** 5816

Werner T, Motyka V, Laucou V, Smets R, Van Onckelen H, Schmülling T (2003) Cytokinin-deficient transgenic *Arabidopsis* plants show multiple developmental alterations indicating opposite functions of cytokinins in the regulation of shoot and root meristem activity. Plant Cell **15:** 2532-2550

Werner T, Nehnevajovà E, Köllmer I, Novák O, Strnad M, Krämer U, Schmülling T (2010) Root-specific reduction of cytokinin causes enhanced root growth, drought tolerance, and leaf mineral enrichment in *Arabidopsis* and tobacco. Plant Cell **22:** 3905-3920

Wu G, Park MY, Conway SR, Wang JW, Weigel D, Poethig RS (2009) The sequential action of miR156 and miR172 regulates developmental timing in *Arabidopsis*. Cell **138:** 750-759

Yamaguchi A, Kobayashi Y, Goto K, Abe M, Araki T (2005) *TWIN SISTER OF FT* (*TSF*) acts as a floral pathway integrator redundantly with *FT*. Plant Cell Physiol **46:** 1175-1189

Yoo SK, Chung KS, Kim J, Lee JH, Hong SM, Yoo SJ, Yoo SY, Lee JS, Ahn JH (2005) *CONSTANS* activates *SUPPRESSOR OF OVEREXPRESSION OF CONSTANS 1* through *FLOWERING LOCUS T* to promote flowering in *Arabidopsis*. Plant Physiol **139:** 770-778

Zhang KW, Novak OR, We, ZY, Gou MY, Zhang XB, Yu Y, Yang HJ, Cai YH, Strnad M, Liu CJ (2014) *Arabidopsis* ABCG14 protein controls the acropetal translocation of root-synthesized cytokinins. Nat Commun **5:** 3274
